# Activity-Based Therapies for Repair of the Corticospinal System Injured during Development

**DOI:** 10.3389/fneur.2014.00229

**Published:** 2014-11-24

**Authors:** Kathleen M. Friel, Preston T. J. A. Williams, Najet Serradj, Samit Chakrabarty, John H. Martin

**Affiliations:** ^1^Department of Neurology, Brain and Mind Research Institute, Weill Cornell Medical College, New York, NY, USA; ^2^Burke Medical Research Institute, White Plains, NY, USA; ^3^Department of Physiology, Pharmacology and Neuroscience, City College of the City University of New York, New York, NY, USA; ^4^School of Biomedical Sciences, Faculty of Biology, University of Leeds, Leeds, UK; ^5^The Graduate Center of the City University of New York, New York, NY, USA

**Keywords:** corticospinal tract, activity-dependent development, motor cortex, motor cortex stimulation, cerebral palsy, mirror movements

## Abstract

This review presents the mechanistic underpinnings of corticospinal tract (CST) development, derived from animal models, and applies what has been learned to inform neural activity-based strategies for CST repair. We first discuss that, in normal development, early bilateral CST projections are later refined into a dense crossed CST projection, with maintenance of sparse ipsilateral projections. Using a novel mouse genetic model, we show that promoting the ipsilateral CST projection produces mirror movements, common in hemiplegic cerebral palsy (CP), suggesting that ipsilateral CST projections become maladaptive when they become abnormally dense and strong. We next discuss how animal studies support a developmental “competition rule” whereby more active/used connections are more competitive and overtake less active/used connections. Based on this rule, after unilateral injury the damaged CST is less able to compete for spinal synaptic connections than the uninjured CST. This can lead to a progressive loss of the injured hemisphere’s contralateral projection and a reactive gain of the undamaged hemisphere’s ipsilateral CST. Knowledge of the pathophysiology of the developing CST after injury informs interventional strategies. In an animal model of hemiplegic CP, promoting injured system activity or decreasing the uninjured system’s activity immediately after the period of a developmental injury both increase the synaptic competitiveness of the damaged system, contributing to significant CST repair and motor recovery. However, delayed intervention, despite significant CST repair, fails to restore skilled movements, stressing the need to consider repair strategies for other neural systems, including the rubrospinal and spinal interneuronal systems. Our interventional approaches harness neural activity-dependent processes and are highly effective in restoring function. These approaches are minimally invasive and are poised for translation to the human.

## Introduction

The corticospinal (CS) system is the principal motor system in humans and many mammals for skilled movements. Damage to this system results in significant motor impairments. In maturity, loss of function after CS system injury, such as weakness and paresis, predominates. Essential coordination necessary for even the simplest of skilled movements, like reaching, grasping, and feeding, is also typically lost. Loss of motor skill and coordination are thought to be due primarily to the loss of the direct projections of the corticospinal tract (CST) from motor cortex to spinal cord motor circuits. CS system damage during development also leads to the gain of aberrant and debilitating functions that are key motor impairments in cerebral palsy (CP) (1). These include hyperreflexia and spasticity as well as aberrant limb and postural coordination. Mirror movements are also common in CP, particularly hemiplegic CP (2). Gain of aberrant functions in CP likely reflects several inter-related processes, including a loss of flexible and individuated muscle control replaced by relatively fixed motor synergies, and hyperreflexia and spasticity (3–6). Our research has identified another important factor contributing to impaired control – development of misprojections between spared cortical motor pathways and spinal and brain stem motor centers. This is maladaptive developmental miswiring of CS motor circuits, both of the injured system and the system that is spared. A critical question that we will focus on in this review is why the loss of CS connections due to perinatal brain trauma leads to maladaptive development of the surviving connections. Whereas it has been well-established that the brain is extraordinarily plastic early in development, beyond that there is a paucity of mechanisms to inform why miswiring occurs and how best to intervene. Our work in animals provides an understanding of the mechanisms underlying miswiring and a strategy to repair abnormal CS connectivity.

In this review, we will first examine normal CS system development, including new findings on genetic developmental cues governing the laterality (i.e., contralateral or bilateral) of CST projections and motor function. Knowledge of the basic underlying genetic mechanisms of CST development can be leveraged to help inform why brain injury in CP can have a profound effect on wiring of the CST and the gain of aberrant functions. Next, we consider the role of neural activity-dependent processes in refinement of the CST from a bilateral to predominantly contralateral motor pathway. In the context of normal development, we briefly consider co-development of the CST and the rubrospinal system, the other major system for limb movement control. Limb movement normally reflects the dual actions of the cortical and rubrospinal systems (7, 8). Although we do not as yet know the specific reaction of the rubrospinal system to damage of the CS system, during the period when CS system damage leads to CP is when the rubrospinal system seems to play a unique role in limb control. Then, we will show how knowledge of normal development informs the question of why particular CST misprojections occur after early brain injury. Finally, we discuss how harnessing activity-dependent synaptic competition, which is key to normal development, leads to restoration of CST connections and recovery of motor skills in an animal model of hemiplegic CP. The aim of this review is to present the mechanistic underpinnings of motor system development derived from animal models, not to summarize therapeutic options for patients with CP. Nonetheless, toward the end of this review, we discuss strategies for neural repair based on our animal model that can be readily implemented in humans because they can be non-invasive or minimally invasive.

## Genetic Factors Help Establish the Laterality of CST Spinal Projections during Development

To understand why the CST develops misprojections in CP and how this relates to the motor impairments, we must examine first the mechanisms of normal development. As for other neural systems, the CST depends on the interplay between genetics, neural activity, and experience to achieve appropriate circuit formation and performance. Genetic mechanisms specify which cortical neurons develop to become CST neurons, and others develop to become interneurons and other projection neurons (9–11). Genetics also plays a key role in guiding CST axons to their targets in the spinal cord. Here, we focus on axon guidance because this is both important for normal wiring of connections and sets the stage for understanding why CST projections go awry after perinatal injury.

Diverse guidance molecules shepherd growing CST axons to their brain stem and spinal targets, including decussation of most CST axons from the medullary pyramid to the contralateral spinal cord white matter (12). CST neurons have complex sets of receptors that enable the guidance molecules present throughout the developing central nervous system to act as ligands to affect steering of the growth cone to ensure that CST axons reach their intended targets (13). The majority of CST axons reach the contralateral spinal segments after decussating in the pyramid and then project extensively into the gray matter on the same side as the descending spinal axons. This is the predominantly contralateral projection, characteristic of normal CST development.

**Figure 1:**
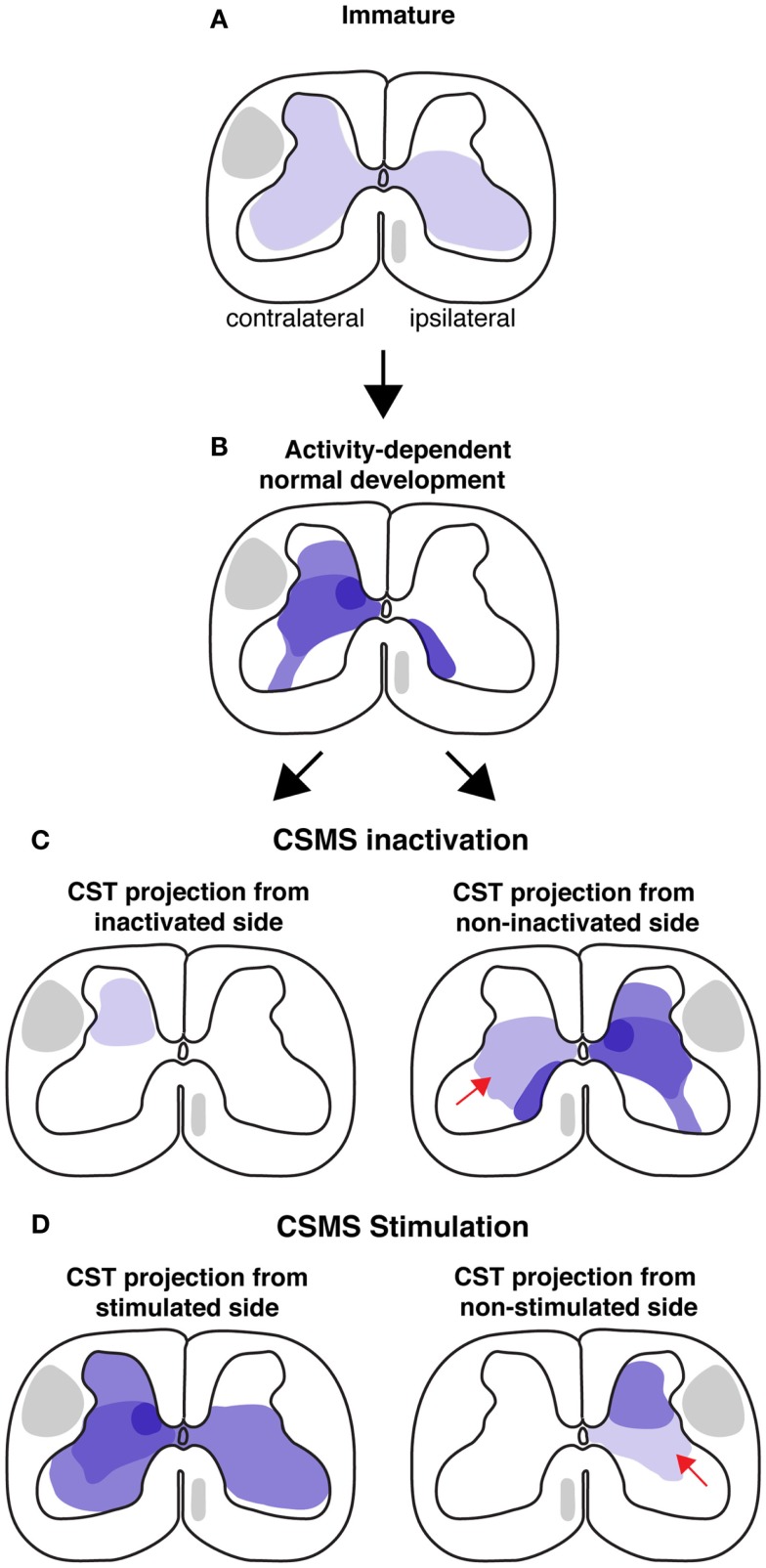
**Schematic illustrations of CST development in cat model during normal conditions and after activity manipulations**. Each illustration is a semi-schematic representation of the CST cervical projection pattern. **(A)** Immature projection. Contralateral and ipsilateral are with respect to the motor cortex of origin of the pathway. The immature pattern is bilateral. **(B)** Normal development results in a predominantly contralateral projection, with multiple sites of high projection density (e.g., intermediate zone). **(C)** CSMS unilateral inactivation results in a greatly contracted termination space (left). The non-inactivated side develops aberrant ipsilateral misprojections. **(D)** Electrical stimulation of the corticospinal motor system (CSMS) results in maintenance of many of the extensive terminations observed in early development, as shown for the stimulated side (left). The non-stimulated side (right) develops fewer ventral projections (arrow).

In the spinal cord, the receptor tyrosine kinase EphA4, along with its ligand EphrinB3, restricts CST outgrowth from the contralateral to the ipsilateral spinal gray matter and thereby helps ensure a predominantly contralateral termination pattern (14–17). EphA4 receptors on the CST growth cone bind to EphrinB3 on midline glial cells, which leads to axonal retraction. Evidence suggests that the overall level of EphA4 is used during development to regulate the amount of recrossing of the CST in the spinal cord and, in turn, the extent to which the developing CST establishes bilateral spinal terminations (17). In genetically normal animals – and likely in humans, as well – this mechanism results in a significant percentage of CST axons that project into the gray matter and recross within the spinal cord early in development. In the cat, which has been studied extensively, early-developing ipsilateral CST projections, which may be as much as 50% of the contralateral projection (18), are subsequently refined to a smaller number that is maintained into maturity (Figure 1). This refinement (discussed further below) establishes the predominantly contralateral CST projection pattern. Similarly, in normal human development, single-pulse transcranial magnetic stimulation (TMS) of motor cortex in neonates evokes bilateral responses, suggestive of strongly bilateral projections, whereas by 6 months of age, only contralateral responses are evoked (19, 20). The amount of recrossing also seems to vary across different animal species, with the monkey showing more than the cat or rodent (21, 22); although this has not been studied systematically.

**Figure 2:**
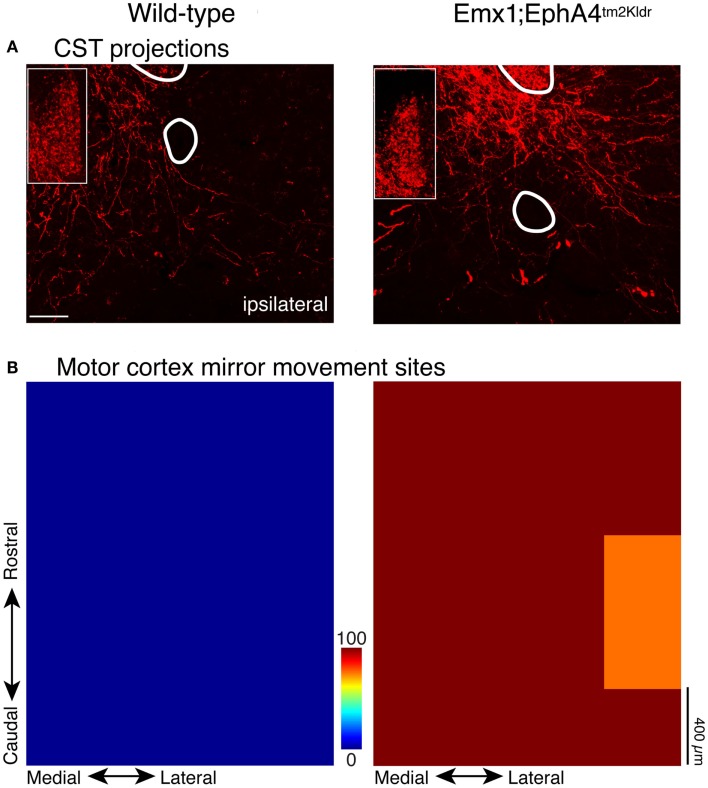
**CST in wild type and EphA4 conditional knockout mouse**. Modified from Serradj et al. (17). **(A)** CST terminations within the intermediate zone flanking the midline. Wild type mice (left) have a predominantly contralateral projection, whereas the mouse with conditional elimination of the EphA4 gene in the forebrain (right) has a strongly bilateral projection. The insets show labeling in the dorsal column, which is the location of the CST in the mouse; labeling is comparable in the two mice. **(B)** Color-coded representations of sites in motor cortex where electrical stimulation evoked bilateral movements (e.g., mirror movements; average of five mice in each category). Whereas no “mirror sites” existed in the wild type mice (left), they were ubiquitous in the mutant mice (right).

Mouse genetics can be leveraged to study the question of development and maintenance of CST laterality, and importantly, inform the functional significance of bilateral CST projections for movement control. When the gene for EphA4 is eliminated selectively in the forebrain of mice, but not in the brain stem and spinal cord, the CST develops a strongly bilateral projection to the spinal cord (Figure 2A). These bilateral CST axons terminate on normally organized spinal circuits within the spinal cord (16, 17). An aberrant bilateral CST projection in mature mutant mice underlies robust bilateral CS motor circuit changes and bilateral voluntary behaviors. With bilateral CSTs, electrical stimulation of the motor cortex evokes bilateral limb muscle responses (16, 17), and a bilateral motor representation in motor cortex (17). In the knockout mouse, we characterized the motor map in terms of the number of sites where stimulation at threshold evoked mirror movements (identical movements of each forelimb). The wild type mice have no sites with this property, while approximately 80% of sites in all knockout mice evoked mirror movements (Figure 2B) (17). This shows that the aberrant bilateral CST projections in maturity are effective in activating spinal motor circuits bilaterally.

**Figure 3:**
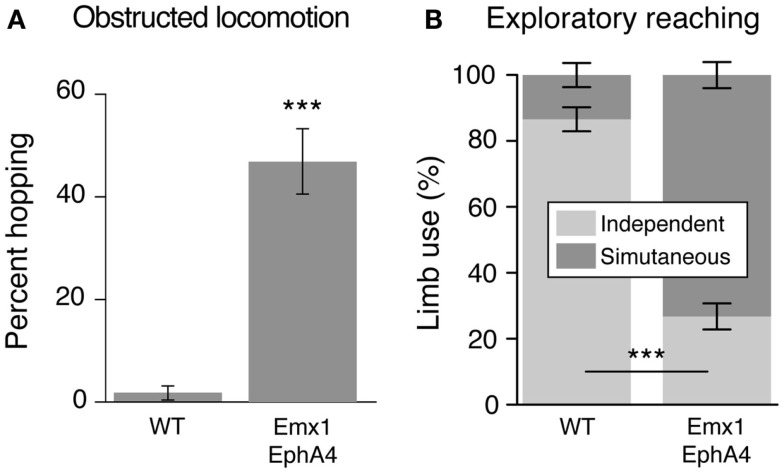
**The conditional EphA4 knockout mouse displays bilateral voluntary fore limb movements**. Modified from Serradj et al. (17). **(A)** Obstructed locomotion. Wild type mice show alternate stepping whereas the EphA4 conditional mutant mice show synchronous fore limb movements (hopping) during obstructed locomotion. **(B)** Exploratory reaching. Wild type mice show independent limb use whereas the EphA4 conditional mutant mice show synchronous limb, or mirror movements, during reaching.

Does development of a bilateral CST and a mirror movement representation in motor cortex produce clear and consistent bilateral voluntary motor responses? This question was addressed by examining two motor behaviors that WT mice produce using unilateral limb movement: obstructed locomotion, where the animal steps over obstacles, and exploratory reaching (Figure 3). Both behaviors are voluntary. In obstructive locomotion, the animal uses obstacle sensory information (e.g., obstacle height and distance) to modify the gait pattern to clear the obstacle (23). And in exploratory reaching, the animal stands on its hind legs and reaches to explore the walls of the enclosure within which it is located (17). WT animals use alternate stepping over the obstacle and reach with one or the other forelimb (Figure 3). By contrast, EphA4 knockout mice rarely use alternate stepping to clear the obstacle, but instead use bilateral synchronous forelimb and hind leg movements, resembling hopping. During exploratory reaching, EphA4 knockout most commonly use both arms simultaneously, resembling mirror movements in people with CP (2). To summarize, by manipulating EphA4 signaling genetically we show the behavioral significance of ipsilateral CST misprojections (17). As we discuss below, ipsilateral CST projections become highly reactive after unilateral brain injury in CP. Similar to the EphA4 knockout mice, with a maladaptive expansion of the ipsilateral CST, many people with CP express mirror movements. Our findings using a genetic model further show that bilateral CST projections, not aberrant bilateral spinal circuits, can explain the presence of mirror movements.

## The Activity of the Motor Systems and Limb Use Refine CST Projection Patterns

Guidance molecules help to establish a coarse early spinal termination pattern of the CST. This pattern is subsequently refined later in development into the mature pattern (Figure 1). What is the mechanism by which the early coarse pattern of connections is refined? Since refinement occurs postnatally as the animal begins to express skilled motor behavior, we focused on the role of activity-dependent processes in establishment of the proper patterns of CST connections with spinal circuits. Our studies in the cat demonstrate an important role for the constitutive level of activity of the CS system in each hemisphere in establishment of spinal connections and a role for limb use, which likely reflects activity patterns. When activity in one motor cortex is blocked pharmacologically by infusing the GABA_A_ agonist Muscimol during an early sensitive period, CST axons withdraw their projections [Figure 1C; left spinal cord; (24)]. Preventing use of one limb during a similar period has a similar effect on development of contralateral CST projections (25). By contrast, when electrically stimulated, CST axons extend more projections (Figure 1D; left spinal cord)(26). This shows the importance of activity-dependent factors in shaping the pattern of CST spinal projections and further suggests interactions – possibly competitive (see below) – between the CS systems from each hemisphere.

The unilateral activity interventions have major bilateral effects. The non-inactivated side (Figure 1C, right spinal cord) develops a normal contralateral projection but, additionally, an aberrant ipsilateral projection (Figure 1C, right, red arrow). Importantly, a bilateral CST from the less-affected side in often considered pathognomonic for hemiplegic CP (discussed below). Similarly, the non-stimulated side develops a diminished projection, with fewer intermediate and ventral projections (Figure 1D; bottom row, red arrow). These findings, together with more limited results from bilateral treatment [activity blockade; (27)], indicates the importance of the relative amount of activity in the developing CS system. We will see below that this is a key finding for understanding the pathophysiological mechanisms underlying CP.

Thus, guidance mechanisms driven by genetic regulation of EphA4 initially help establish the density of ipsilateral CST axons. Activity- and use-dependent processes subsequently shape the pattern initially established by genetic mechanisms. A plausible developmental strategy is that the more liberal the recrossing of CST axons, the greater opportunity for bilateral connections to be supported by early-developing bimanual limb movements. For example, if the developing child does not engage in extensive bimanual control, then more ipsilateral CST projections are eliminated compared with a child that uses more bimanual control. Further, the animal data provide support for a developmental rule whereby the more active (and more extensively used) connections of the developing CS system overtake less active/used connections. This helps explain the normal development of a predominantly crossed CST and the elimination of the early ipsilateral projections. As we discuss below, this activity-based rule also helps explain misprojections in CP.

## Motor Representation Development in Motor Cortex and the Role of the Red Nucleus in Early Movement Control

The CST is one of many motor systems of the brain and spinal cord (28). In maturity, the CST functions together with the red nucleus (RN), which gives rise to the other major descending pathway for limb control, the rubrospinal tract (7, 8). The CS system is thought to play a greater role in more flexible and adaptive movements, and the rubrospinal system, in more automatic limb movements. Whereas the rubrospinal system has been speculated to play a role in recovery after CST damage in maturity (29, 30), its role in motor development in health and disease is not yet known (4). Interestingly, the rubrospinal system may have even more anatomical and functional prominence early in human development than it does later in life (31). We have investigated development of the rubrospinal system in relation to CST development.

**Figure 4:**
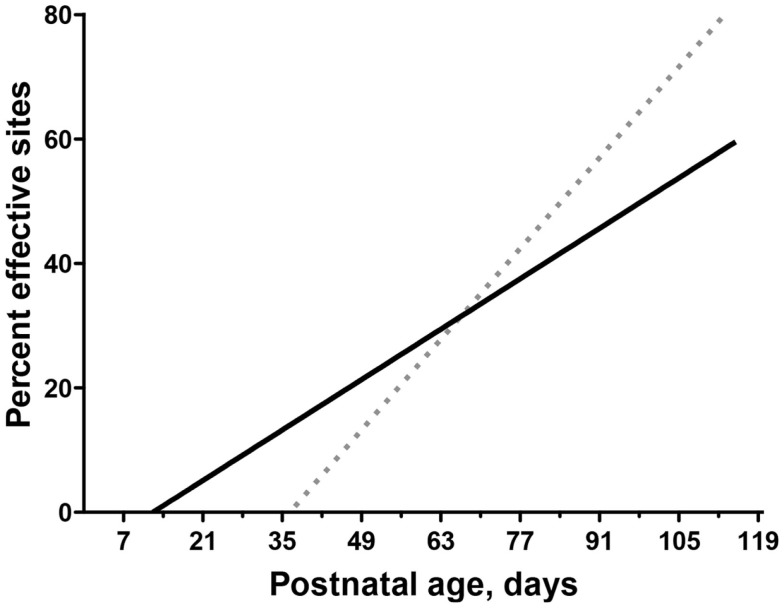
**Differential motor map development in motor cortex and red nucleus**. Modified from Williams et al. (32) and Chakrabarty and Martin (35). Comparison of age-dependent increases in the percentage of sites in motor cortex (dotted line) and RN (solid line) where stimulation produced a contralateral motor response (termed effective sites). The linear fit is shown for both motor cortex (*Y* = 1.54*x* − 68.62) and RN (*Y* = 0.66*x* − 10.03).

When the developing CS and rubrospinal systems are directly compared in an animal model, it has been shown that the rubrospinal system matures earlier than the CST (32). Early behavioral contributions of the CS and rubrospinal system can be evaluated by comparing development of their motor representations. The motor cortex motor map is a good indicator of CS system function (33). In the cat, for example, the motor cortex map comes “online” at about postnatal week 7 (Figure 4), dotted regression line; (34, 35) and this is when several measures of voluntary movement control – including object manipulation, and social/play interactions – develop (36, 37). Map development plateaus at about postnatal week 12, and this is also when expression of voluntary control stabilizes. Surprisingly, development of the red nucleus motor map begins earlier than the motor cortex motor map (Figure 4). In motor cortex, proximal limb joints are represented at younger ages then distal joints, also paralleling late development of distal skills (35). By contrast, in the red nucleus distal as well as proximal forelimb muscles are represented at the outset. Taken together, these findings suggest that the rubrospinal tract is important for establishing the rudiments of motor skills before the CST has come online.

An important question, yet to be resolved, is the extent to which these two motor systems interact during development. Emerging evidence from our laboratory suggests that there is an activity-dependent interaction between the developing corticorubral and rubrospinal projections (38, 39). When motor cortex activity is blocked during early development, the red nucleus on the side of the inactivation may have an enhanced development, while development of the red nucleus motor map on the opposite side is remarkably impaired. This finding hints at a competition between the developing rubrospinal and CS systems. Red nucleus compensation on the injured side could be part of the biological basis of partial recovery after developmental cortical injury. That the rubrospinal system precedes CS system development suggests that it may not be as prone to the same miswiring as the CST following early activity manipulations because its sensitive period is earlier.

## Mechanisms of Maladaptive Development of the Corticospinal System after Unilateral Injury

As expected, cortical and white matter injury destroys the cells of origin and connections of the CS system. In doing so, injury will not only disrupt CST function but system-wide development as well. With a predominantly unilateral injury, what is not well understood is why damage on one side leads to development of misprojections of the spared CST from the uninjured hemisphere. Our studies strongly suggest that the same activity-dependent mechanisms that ensure optimal CST development under normal conditions can become maladaptive after perinatal injury to the CS system.

**Figure 5:**
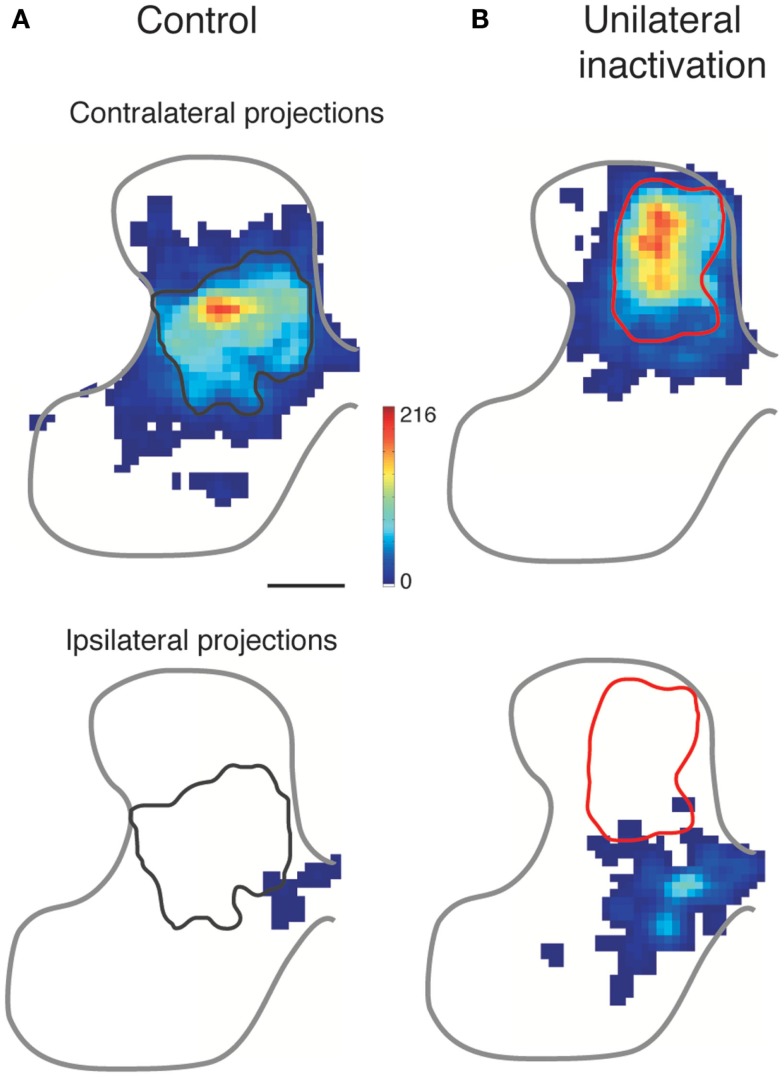
**Effect of activity blockade on CST development**. Modified from Friel and Martin (24). Color-coded representations show local CST density in the cervical gray matter. The normal contralateral CST projection **(A)** is densest to the intermediate gray matter, and does not overlap with the ipsilateral projection from the other hemisphere, which is located medially. After perinatal inactivation **(B)**, the contralateral projection shifts dorsally and the ipsilateral projection from the other hemisphere expands laterally. The gray and red outlines mark the densest territory of contralateral CST projections for normal development (left column) and after unilateral CST inactivation (right column), respectively. The contralateral and ipsilateral “heat maps” are plotted with the same color scales.

With an initial bilateral organization, the typical-developing CST eliminates most ipsilateral spinal projections (Figure 1). As discussed above, the initial density of ipsilateral CST projections is regulated, in part, by a genetic mechanism (EphA4) and the subsequent reduction in ipsilateral projections reflects an activity-dependent refinement process (12). Figure 5A shows the normal contralateral (top) and ipsilateral (bottom) projections. When the activity of the CS system in one hemisphere is reduced, by intracortical infusion of muscimol, the affected hemisphere is not able to establish its normal projection pattern contralaterally (Figure 5B, top). In a reciprocal manner, the ipsilateral projections of the CS system in the other hemisphere are better able to compete with the less active CST for synaptic connections with spinal cord neurons (Figure 5B, bottom). In these experiments, CST axons from only a small portion of the forelimb area of motor cortex were labeled. Nevertheless, comparison of the control and inactivated animal revealed a 3.6-fold increase in the spatial extent of the ipsilateral projections with a peak approximately 1/3 that of the contralateral projection. Thus, the active CS system maintains, and likely strengthens, its early bilateral organization (Figure 5B, bottom). The incursion of abundant CST outgrowth into the ipsilateral spinal gray matter greatly impedes development of the less active CST. The contralateral axons of the less active CST are prevented from typical outgrowth and synapse formation (24, 40). Further, there is a dorsal shift for the inactivated CST (24, 40, 41). We do not yet know the mechanism of this circuit change. However, it is apt to be functionally significant because this dorsal area of the dorsal horn is more concerned with the processing of somatic sensory information than motor output (28). Because of this location, the impaired CST may have reduced access to spinal motor circuits and, as a consequence, is much less functional. The question remains in the animal model if this aberrant dorsal CST projection is maladaptive. By not targeting their proper spinal circuits, this projection could underlie aberrant contralateral control, such as spasticity, incoordination, and reflex radiation.

**Figure 6:**
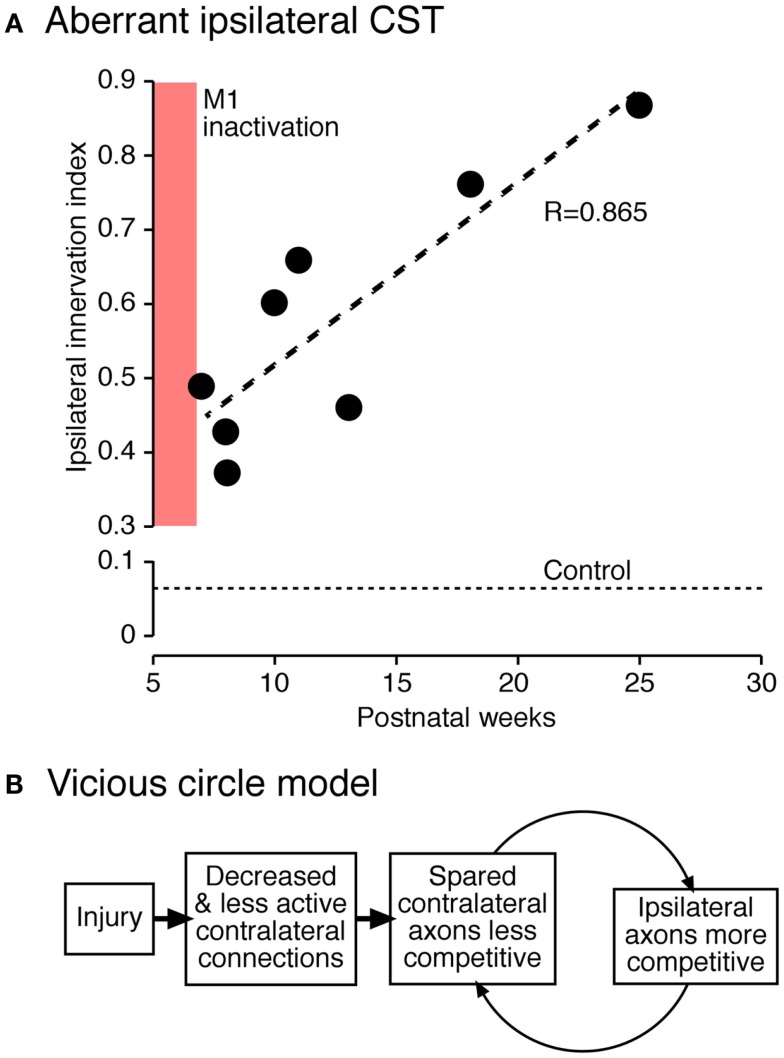
**Vicious circle hypothesis (42)**. **(A)** Aberrant ipsilateral corticospinal terminations further develop postnatally. The graph plots the ratio of local density of ipsilateral and contralateral CST terminations. **(B)** Hypothesis of progressive decline of less active connections and complementary augmentation of more active connections.

As development progresses, the ipsilateral CST from the active side continues to establish connections at the expense of the contralateral projection from the inactive side. We showed that this process progresses even after activity is restored to the previously silenced side (Figure 6). We propose that perinatal injury before elimination of early ipsilateral CST projections creates a “vicious circle” of further loss of injured and gain of spared CST fibers (42). This is due to impaired capacity of the injured side to maintain connections, and a concomitant robust reactive increase in ipsilateral CST projections from the undamaged hemisphere. The underlying mechanism may be activity-dependent synaptic competition, with a loss of competitive ability of the injured side and a gain of competitive ability by the undamaged (or less damaged) side.

We equate the less active CST in our model with the damaged CST after a lesion. The affected limb is used less than normal, thus the surviving fibers of the CST serving that limb would be expected to be less active. There also would be reduced afferent feedback because of the paucity of movements, and possibly reorganization of proprioceptive inputs to the spinal cord (43, 44). Conversely, the unaffected (or less affected) limb is used more and the undamaged CST would be expected to be more active. Thus, experimentally manipulated activity changes both inform mechanism (e.g., activity may be more important than the physical loss of connections due to damage) and are also a reasonable model for what happens after a unilateral lesion. Damage to one hemisphere, and the associated lack of limb use, can thus lead to a dual vulnerability. The damaged side loses much of its contralateral connections to injury, while the undamaged (or less damaged) side “fills in” these denervated territories with ipsilateral misprojections and further constrains development of the remaining contralateral CST projections.

We hypothesize that the aberrant, and possibly maladaptive, ipsilateral CST in people with hemiplegic CP reflects a basic mechanism for synaptic stabilization that goes awry after injury. With injury, more robust than loss of activity as in our animal model, the loss of connections and lack of affected limb use makes this side less able to compete for synaptic connections with spinal motor circuits than the uninjured system. By contrast, the less-affected CST concomitantly gains synaptic competitive ability. When damage occurs after the early ipsilateral CST projections are eliminated, reduced synaptic competition may continue to play an important role in the evolving motor impairment. In maturity, loss of connections of the damaged system is counterbalanced by a small reactive increase in spared ipsilateral CST projections from the undamaged hemisphere (45, 46) and a reactive increase in proprioceptive afferent projections (47, 48). At this point after an injury in maturity, denervated spinal circuits would be driven more by afferent fibers and ipsilateral CST projections. This too likely results in motor impairment.

## Interventional Strategies

Knowledge of the pathophysiology of the developing CST after injury informs interventional strategies for protecting the damaged system from further loss of connections and function during development. This knowledge is also helpful in devising strategies for repairing aberrant CS circuits once development has taken place. As discussed, a well-known misprojection of the CST in patients with hemiplegic CP is development of ipsilateral misprojections. Eyre et al. (49) have shown that perinatal injury results in the progressive loss of the damaged CST and maintenance and possible strengthening of the ipsilateral CST; this is akin to the vicious circle discussed above (42). The net outcome is that the spared side develops an aberrant bilateral CST. We infer from our genetic model [Figures 2 and 3; (17)] that one consequence of these bilateral misprojections in CP is mirror movements (2). Other misprojections are likely to play a role in impaired intra-limb coordination, reflex radiations, and spasticity. We hypothesize that during the period when the ipsilateral projections are strengthening after perinatal injury, activity-dependent competition is “out of control” driving down contralateral function of the affected side and establishing stronger ipsilateral projections from the non-affected side.

Using the cat model described in the previous section, we aimed to repair the aberrant pattern of connections and restore function by harnessing activity-dependent processes. According to our competition hypothesis, either improving the capacity of the injured system to compete for spinal synaptic connections or diminishing the unaffected system to compete, should help restore a more normal CST pattern of connections and improve function. We tackled both approaches and achieved differential success that can be explained on the basis of a dosing effect.

**Figure 7:**
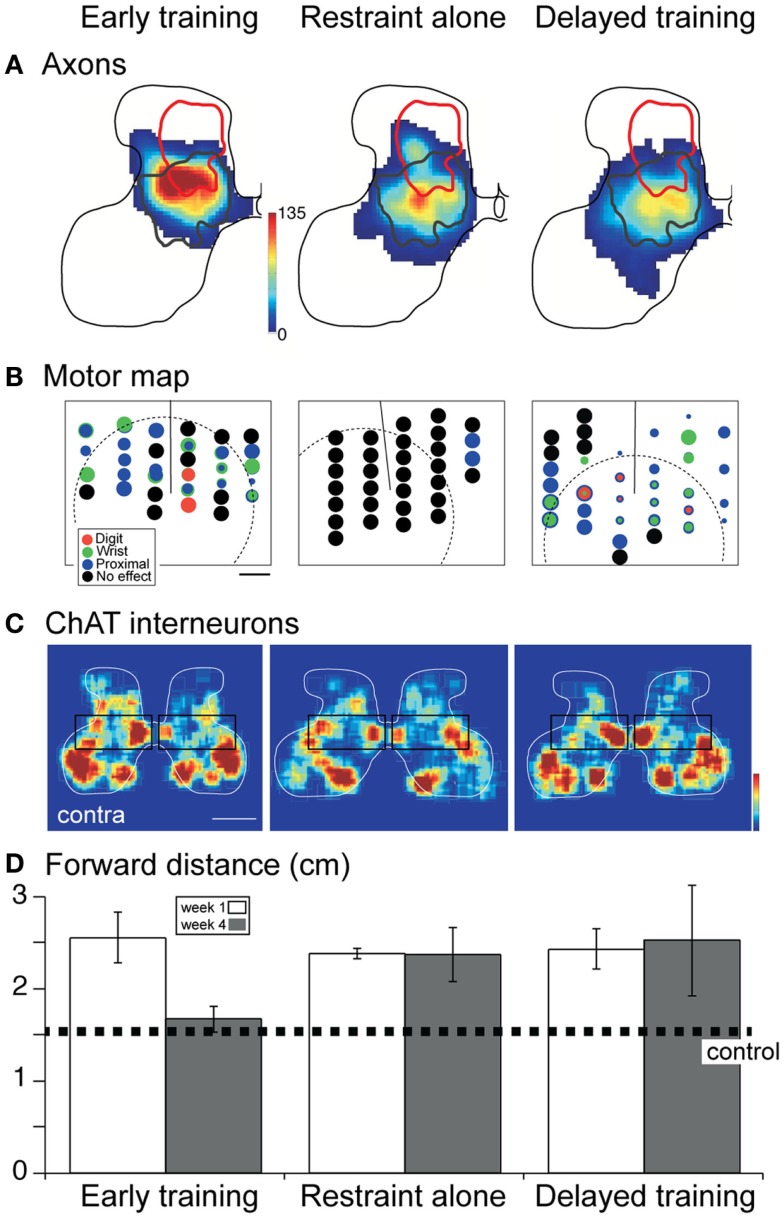
**Effect of constraint of the unimpaired limb on CST projections, the motor cortex motor map, spinal cholinergic interneurons, and skilled behavior**. Modified from Friel et al. (41). **(A)** Distribution of CST projections in the cervical enlargement. Density of projections is represented by a color scale, with red corresponding to the densest projections. All “heat maps” are plotted with the same color scale. The red line corresponds to the aberrant dorsal projection of the inactivated CST (as shown in Figure 6), whereas the dark gray line is the normal distribution (Figure 6). **(B)** Motor cortex motor map. Each site examined for a motor response with stimulation is marked by a circle. The threshold for evoking a contralateral motor response is represented inversely by circle diameter. Black corresponds to no response sites. **(C)** Color-coded representations of local cholinergic interneuron density. Boxes highlight differences in relative density. Contralateral compared with ipsilateral density is greatest for early training. **(D)** Forward distance is a measure of reaching accuracy. The dotted line marks normal performance. Over-reaching is characteristic at 1 week for all groups. Only the early trained group shows recovery to normal distances; performance of the other groups remains errant.

In two separate studies, we showed that promoting the activity of the injured system (26) or decreasing the activity of the undamaged system (24) immediately after the period of injury leads to partial repair of the CST and restoration of skilled motor function. These remarkably effective activity-based interventions provide proof-of-principle of the capacity to harness activity after developmental impairment to repair CST connections and function. We next took a more clinically minded approach and used constraint of the less-impaired limb at two time points, with and without behavioral training, to determine if behavioral approaches are similarly effective. We examined three conditions: (1) constraint immediately after the impairment; (2) constraint immediately after the impairment plus intensive motor training (reach to grasp); and (3) delayed constraint with intensive motor training. It is important to recognize that the effects of early developmental impairment [e.g., Figure 6B; CST abnormalities and motor control impairments; (24, 50)] are permanent without further intervention. Remarkably, whereas all three interventions result in comparable repair of the contralateral CST (Figure 7A), there were differences in the motor cortex motor maps and spinal interneuronal function that accounted for whether or not animals showed behavioral recovery.

With constraint and intensive training both initiated early in development (Figure 7), there was restoration of the motor cortex motor map; a complete representation of contralateral forelimb joints was present (Figure 7B) (41). Thus, representational plasticity, which occurs throughout the animals lifetime (35, 51), helps to provide an effective substrate for contralateral limb control. Although the absolute numbers of spinal cholinergic interneurons were not different, side-to-side, we also observed relative increases in spinal cholinergic interneurons (Figure 7C; boxed region compares interneurons in the intermediate zone). These interneurons may play a key role in relaying CST signals to motoneurons (52–55). Together, the motor map and interneuronal changes support behavioral recovery. Without intensive training (Figure 7, middle column), the motor cortex motor map failed to show representational plasticity; the motor map was essentially lost (41). Further, interneuronal numbers did not improve. Together, the absence of the map and insufficient interneuronal changes could have prevented behavioral recovery. Clearly, restraint alone is not therapeutic; it needs to be combined with training.

Importantly, delayed intervention into the feline equivalent of young adolescence led to restoring the motor map but not cholinergic spinal interneurons [Figure 7, third column; (41)]. Since this animal group did not recover function, but did show repaired CST projections and M1 motor map, it shows the importance of spinal circuitry and possibly other pathways (e.g., rubrospinal tract) in normal and recovered function. In the cat, spinal cholinergic interneurons develop during the same period when the CST connections are being refined (53). We hypothesize that there is a critical period for spinal cholinergic interneuron development, under CST activity control (41). The capacity to restore this interneuronal function is restricted to the early intervention period. This demonstrates the importance of repair strategies that take other neural systems into account. The rubrospinal system is another potentially important system that should be considered in repair after perinatal damage.

Several studies have demonstrated efficacy of activity-based therapies for children with hemiplegic CP (56–61). These therapies – either constraining the less-affected upper limb and intensively training the affected upper limb, or intensively training bimanual upper limb movements – improve hand function in children with hemiplegia. Since intensive therapy requires children to engage and follow instructions, these therapies are typically delivered to school-aged children. It is hypothesized that, just as in our cat model, these therapies increase activity of the impaired side, thus increasing the competitive strength of the impaired side against the less-impaired side. Although these activity-based therapies improve upper limb control, function is not fully restored. Hand function is still most often substantially impaired compared with typically developing children. Our studies in the cat model indicate that early intervention, before the less-impaired CST secures a strong competitive advantage over the impaired CST, may be necessary for mitigating the motor dysfunction of CP.

Although clinical application of activity-based therapies is challenging in young children, there have been efforts to translate therapy to this population. Gordon et al. (62) have developed a caregiver-driven bimanual therapy for toddlers that significantly improved bimanual hand use and performance of functional tasks. Several studies have indicated that enriched environments that encourage infants to move and reach can improve motor outcomes in very young children who have, or are at risk of developing, CP (63–65).

Non-invasive brain stimulation may be a viable strategy for balancing activity-dependent competition between the two sides of the brain after unilateral brain injury. Repetitive transcranial magnetic stimulation (rTMS) has been shown to boost the amount of motor improvements seen after gait or hand training (66, 67). Recently, transcranial direct current stimulation (tDCS) has emerged as a preferred method of non-invasive brain stimulation due to its excellent safety profile, portability, and low cost. tDCS has been tested in a small number of children with hemiplegia, and has been shown to be safe and tolerable (66). Non-invasive brain stimulation may emerge as a potential therapy for very young children, to balance interhemispheric competition before the damaged side loses its foothold in the developing motor system. However, safety is an important concern that must be evaluated further. The use of brain modeling is likely to be an important step in determining the safety of brain stimulation in young children (66, 68).

## Perspectives and Conclusion

We envisage two distinct periods of intervention, where repair of the damaged CS system can occur. Very early during development, during the so-called critical period, activity-based approaches can have a neuroprotective effect by enhancing the ability of the damaged CST to establish spinal connections. This is likely the most effective period since it is directed to facilitate spinal circuit development. Extrapolating from the cat data, in the human this would correspond to the first 6–12 postnatal months (19). Later in development, after aberrant CST connections are established and become permanent if not treated further, there is still a robust capacity to repair. CST growth to original targets and partial elimination of aberrant ipsilateral misprojections can occur. Indeed, intact CST axons are capable of sprouting into the denervated side of the spinal cord in maturity (45, 46, 69). However, the efficacy is strongly reduced after the critical period and is insufficient to restore significant function unless promoted. There also is robust representational plasticity of the motor cortex (41). Importantly, in our model, behavioral therapies do not appear to be effective in repairing aberrant spinal interneuronal circuits later in development. It is plausible that direct activity manipulations, such as motor cortex stimulation (45, 46), are needed to coax spinal interneurons to improve function.

A critical question is if the ipsilateral CST after unilateral development lesion can be adaptive? By themselves, ipsilateral CST projections target different sets of spinal cord laminae compared with their contralateral counterparts (45, 69). As a consequence, different somatic sensory and motor circuits will be engaged by the ipsilateral and contralateral tracts. This points to limitations in function but not necessarily maladaptive functions. Further, the ipsilateral projection in concert with the contralateral projection leads to bilateral control, and impairments such as mirror movements. However, it has been shown in maturity that strengthening spared ipsilateral CST axons actually leads to functional improvements in a rat model of unilateral CST lesion (46, 70). We think that these findings point to a different culprit. It is not the ipsilateral CST that is maladaptive but rather the consequential loss of the contralateral projection due to reduced ability to compete for synaptic connections in the spinal cord. Furthermore, if the loss of the contralateral projection also triggers afferent fiber sprouting during development, as it does in maturity (48), then there are additional potential sources of maladaptive spinal inputs. It may be necessary to repair multiple neural systems – CST, spinal interneurons, afferent fibers, and rubrospinal fibers, to name four – before motor function can be fully restored. Our interventional approaches that harness neural activity-dependent processes are highly effective in an animal model of hemiplegic CP. These approaches are minimally invasive and are poised for translation to the human.

## Conflict of Interest Statement

The authors declare that the research was conducted in the absence of any commercial or financial relationships that could be construed as a potential conflict of interest.
